# The diagnostic challenge of mediastinal sarcoidosis accompanying esophageal cancer

**DOI:** 10.1186/1477-7819-8-15

**Published:** 2010-03-12

**Authors:** Matthias Schauer, Joerg Theisen

**Affiliations:** 1 Department of General Surgery, Heinrich Heine University, Moorenstrasse 5, 40225 Duesseldorf, Germany; 2 Department of Surgery, Technische Universitaet Muenchen, Ismaninger Straße 22, 81675 Munich, Germany

## Abstract

The primary staging of an oesophageal cancer can be difficult, if accompanied by sarcoidosis. In these patients endosonography, CT and PET may not be sufficient for staging purposes concerning lymph node and distant metastases. In these special cases operative biopsies of enlarged lymph nodes and unclear pulmonary nodules have to be obtained. In connection with the radiographic examinations the histopathological results of the biopsies contribute to further precise staging and help to decide on a curative versus a palliative therapy concept.

## Background

The incidence of sarcoidosis averages 1:10.000 in the western world [1]. A risk analysis of cancer from cohorts of Swedish patients with sarcoidosis showed, that the overall relative risk for cancer development is increased, especially the risk for cancer of the lung, stomach, small intestine, liver and skin [2]. The coincidence of sarcoidosis and oesophageal cancer is a rare event. Up until now five such cases were mentioned in the international literature [2].

The documented cases describe the limitations of the possible staging procedures in patients with a thoracic neoplasia accompanied by sarcoidosis. Moreover, a feasible approach towards these cases is being proposed.

## Case presentation

In the year 2007, 283 patients with an adenocarcinoma of the gastro oesophageal junction (AEG) or an oesophageal cancer were admitted to our hospital. Two patients, aged 55 and 66, presented with an oesophageal cancer, a thoracic lymphadenopathy and sarcoidosis in their past medical history.

**Figure 1:**
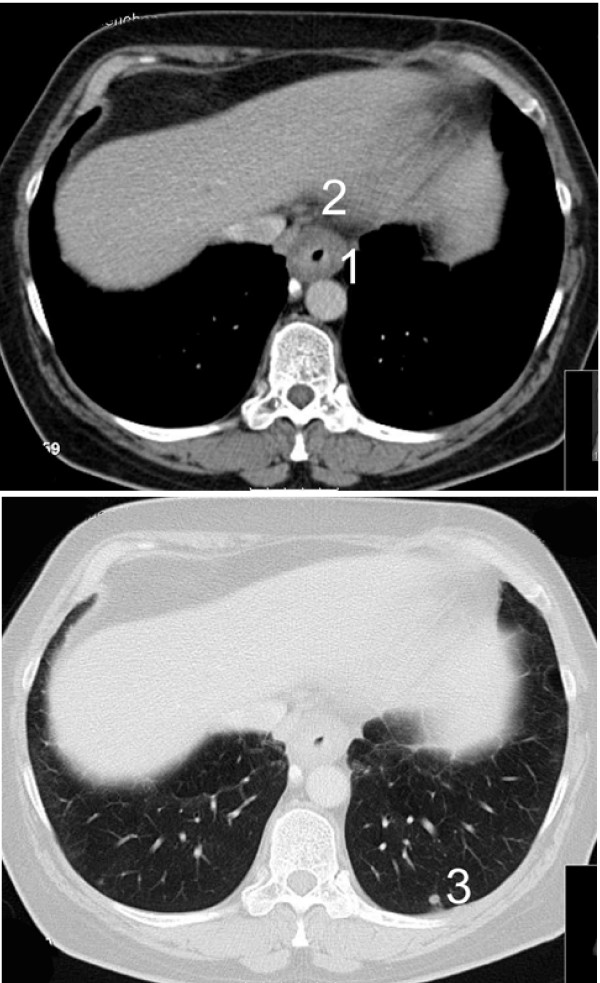
**CT scan showing a circular oesophageal cancer (1.) with paraoesophageal lymph node involvement (2.) and small sarcoid lesions of the lung (3.)**.

**Figure 2:**
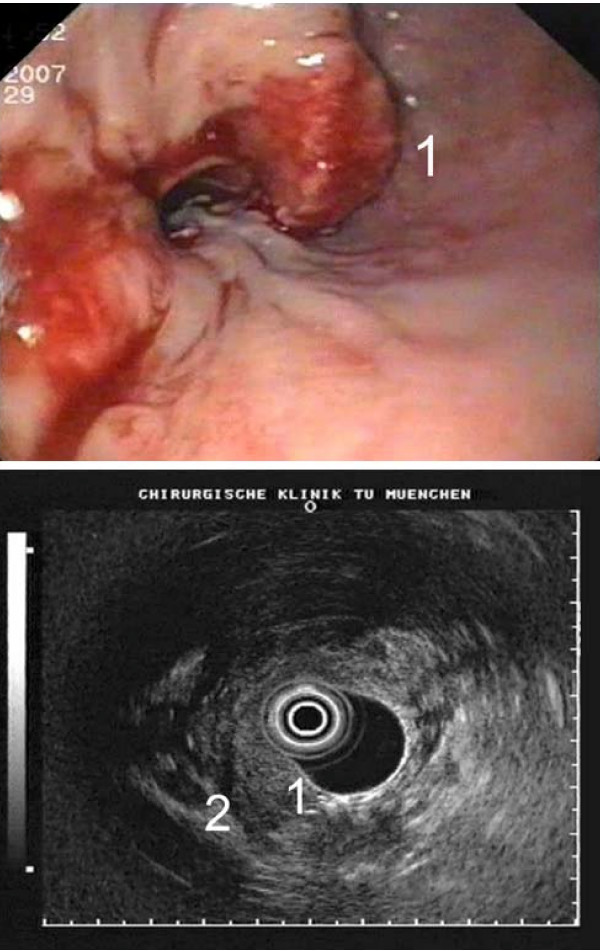
**Endoscopy and endosonography of the oesophageal tumor with infiltration of all mucosal layers (1) and peritumoral lymph nodes (2)**.

The 55-year-old female patient with an adenocarcinoma of the cardia was referred to our hospital after neoadjuvant chemotherapy with cisplatin and 5-FU in Montenegro for a second opinion concerning the resectability of the tumour. In the past medical history a clinical unapparent sarcoidosis was known for the last ten years. The actual CT scan, the endoscopy and the endosonography showed an extensive tumour growth involving the oesophago-gastric junction and enlarged paraoesophageal, perihilar and paratracheal lymph nodes. Multiple small disseminated lesions of the lungs were unchanged compared to the initial CT scan half a year before (Figure 1 and 2). The parenchymal abdominal organs did not show any metastases.

Since CT scan and FDG PET could not differentiate between oesophageal metastases and sarcoidosis of the lungs and the lymph nodes, a mediastinoscopy was performed for further information. Biopsies of the peritracheal lymph nodes showed a granulomatous inflammation with necrosis, consistent with sarcoidosis. Thus an oesophagectomy and a reconstruction with a gastric tube were performed. The histopathological examination showed a complete resection of the cardia cancer with 13 positive locoregional lymph nodes. (UICC-Classification pT_3 _N_2 _(13/68) M_0 _R_0 _G3). The sarcoidosis could be diagnosed in the resected mediastinal, perihilar and truncular lymph nodes. The patient was discharged 17 days after resection.

The second patient, a 66-year old woman with sarcoidosis in the past medical history and a squamous cell cancer of the oesophagus, showed an oesophageal cancer with enlarged paraoesophageal and cervical lymph nodes on CT scan. For further differentiation between sarcoidosis and lymph node metastasis again mediastinoscopy was performed. Histology showed sarcoidosis of the mediastinal and cervical lymph nodes. Because distant metastases could be ruled out a neoadjuvant radio-chemotherapy was performed. The pathologic specimen after transthoracic oesophagectomy showed a good response rate of the squamous cell cancer (UICC-Classification ypT_3 _N_1_(2/17) M_0_R_0 _G3). The resected lymph nodes in the upper mediastinum showed a granulomatous inflammation consistent with sarcoidosis. Both patients participate in our follow-up program for two and a half years already.

## Conclusions

The relative risk for malignant disease in patients with sarcoidosis is increased. Currently two explanations for this phenomenon are discussed in the literature. In patients with a history of sarcoidosis chronic inflammation was suggested to be the putative mediator for the increased cancer risk [2]. In patients with sarcoid like lesions occurring in the draining lymph nodes of tumours after chemo- or radiotherapy the aetiology of the sarcoid reactions is postulated to be an induced T-cell-mediated host response to soluble antigenic tumour factors. The antigenic factors may be either shed by the tumour cells or released during tumour necrosis [2,3]. In our two cases the sarcoidosis was already known before oesophageal cancer was diagnosed, which makes the first of the two hypothesis more possible in these specific cases.

Oesophageal cancer with distant metastases should be treated in a palliative concept without tumour resection. Therefore, the differential diagnosis of distant enlarged lymph nodes (M1a (lymph)) and unclear pulmonary nodules are crucial for the further treatment.

However, the preoperative staging examinations with endoscopy, endosonography, CT- and PET scan is limited and can differ from the postoperative histopathological examination [4]. The dilemma of lymph node diagnosis becomes a specific problem in patients with a previous history of sarcoidosis [5,6]. Even with an additional mediastinoscopy the problem cannot completely be solved since specific lymph node diagnostic can only be done for a small subset of lymph nodes. By including such patients in neoadjuvant protocols this preoperative therapy may help in distinguishing sarcoid like lymph nodes from true metastatic nodes. However, only the postoperative histological examination of the resected specimen can give a precise staging like in our patients.

As FDG-PET and CT scan is used extensively in oncology, clinicians should be aware of sarcoidosis, which can have the same appearance as diffuse metastases. In patients under otherwise good healthy conditions, who could be treated aggressively with a neoadjuvant therapy followed by resection of the tumour in curative intention, pathological diagnosis for exact pretherapeutic staging should be obtained.

## Competing interests

The authors declare that they have no competing interests.

## Authors' contributions

MS reviewed patients' charts, collected radiographic and endoscopic material and drafted the manuscript. JT participated in the design of the study, gave intellectual input, read, corrected and approved the manuscript.

## Consent

Written informed consent was obtained from the patients for publication of this case report and accompanying images. A copy of the written consent is available for review by the Editor-in-Chief of this journal.
